# Prognosis of critically ill immunocompromised patients with virus-detected acute respiratory failure

**DOI:** 10.1186/s13613-023-01196-9

**Published:** 2023-10-13

**Authors:** Guillaume Dumas, Maxime Bertrand, Virginie Lemiale, Emmanuel Canet, François Barbier, Achille Kouatchet, Alexandre Demoule, Kada Klouche, Anne-Sophie Moreau, Laurent Argaud, Florent Wallet, Jean-Herlé Raphalen, Djamel Mokart, Fabrice Bruneel, Frédéric Pène, Elie Azoulay

**Affiliations:** 1 Service de Médecine Intensive-Réanimation, CHU Grenoble-Alpes; Université Grenoble-Alpes, INSERM U1300-HP2, Grenoble, France; 2 Medical Intensive Care Unit, Saint-Louis Teaching Hospital, AP-HP, Paris, France; 3 ECSTRA Team, Biostatistics and Clinical Epidemiology, UMR 1153 (Center of Epidemiology and Biostatistics Sorbonne Paris Cité, CRESS), INSERM, Université de Paris, Paris, France; 4 Nantes Université, CHU Nantes, Médecine Intensive Réanimation, 44000 Nantes, France; 5 Medical Intensive Care Unit, La Source Hospital, CHR Orleans, Orleans, France; 6 Medical Intensive Care Unit, Angers Teaching Hospital, Angers, France; 7 Service de Médecine Intensive et Réanimation (Département R3S), Sorbonne Université, INSERM, UMRS1158 Neurophysiologie Respiratoire Expérimentale et Clinique, and AP-HP, Groupe Hospitalier Universitaire APHP-Sorbonne Université, Site Pitié-Salpêtrière, 75013 Paris, France; 8 Medical Intensive Care Unit, CHU de Montpellier, Montpellier, France; 9 Service de Réanimation Polyvalente, CHRU de Lille - Hôpital Roger Salengro, Lille, France; 10 Medical Intensive Care Unit, Hospices Civils de Lyon, Hopital Edouard Herriot, Lyon, France; 11 Intensive Care Unit, Lyon Sud Medical Center, Lyon, France; 12 Department of Anesthesia and Critical Care, Necker Hospital, Paris, France; 13 Intensive Care Unit, Institut Paoli Calmettes, Marseille, France; 14 Medical Intensive Care Unit, Andre Mignot Hospital, Versailles, France; 15 Medical Intensive Care Unit, Cochin Hospital, Hôpitaux Universitaires Paris Centre, AP-HP, Paris, France; 16 Institut Cochin, INSERM Unité 1016/Centre National de La Recherche Scientifique (CNRS) Unité Mixte de Recherche (UMR) 8104/Université de Paris, Paris, France

**Keywords:** Immunocompromised, Respiratory virus, Influenza, Pneumonia, Mechanical ventilation

## Abstract

**Background:**

Acute respiratory failure (ARF) is the leading cause of ICU admission. Viruses are increasingly recognized as a cause of pneumonia in immunocompromised patients, but epidemiologic data are scarce. We used the *Groupe de Recherche en Réanimation Respiratoire en Onco-Hématologie*’s database (2003–2017, 72 intensive care units) to describe the spectrum of critically ill immunocompromised patients with virus-detected ARF and to report their outcomes. Then, patients with virus-detected ARF were matched based on clinical characteristics and severity (1:3 ratio) with patients with ARF from other origins.

**Results:**

Of the 4038 immunocompromised patients in the whole cohort, 370 (9.2%) had a diagnosis of virus-detected ARF and were included in the study. Influenza was the most common virus (59%), followed by respiratory syncytial virus (14%), with significant seasonal variation. An associated bacterial infection was identified in 79 patients (21%) and an invasive pulmonary aspergillosis in 23 patients (6%). The crude in-hospital mortality rate was 37.8%. Factors associated with mortality were: neutropenia (OR = 1.74, 95% confidence interval, CI [1.05–2.89]), poor performance status (OR = 1.84, CI [1.12–3.03]), and the need for invasive mechanical ventilation on the day of admission (OR = 1.97, CI [1.14–3.40]). The type of virus was not associated with mortality. After matching, patients with virus-detected ARF had lower mortality (OR = 0.77, CI [0.60–0.98]) than patients with ARF from other causes. This result was mostly driven by influenza-like viruses, namely, respiratory syncytial virus, parainfluenza virus, and human metapneumovirus (OR = 0.54, CI [0.33–0.88]).

**Conclusions:**

In immunocompromised patients with virus-detected ARF, mortality is high, whatever the species, mainly influenced by clinical severity and poor general status. However, compared to non-viral ARF, in-hospital mortality was lower, especially for patients with detected viruses other than influenza.

**Supplementary Information:**

The online version contains supplementary material available at 10.1186/s13613-023-01196-9.

## Background

The number of immunocompromised patients is increasing steadily [1]. This is primarily the result of major therapeutic advances that have resulted in an improvement in survival and quality of life in patients with solid tumors, hematological malignancies, solid organ transplants, and various types of auto-immune and auto-inflammatory disorders [2]. However, these patients can encounter several complications which may warrant intensive care unit (ICU) admission [3]. Among them, acute respiratory failure (ARF) is the leading cause of ICU admission with high reported case-fatality [4, 5]. Despite important advances [6–8], ARF remains a challenging clinical situation for clinicians, both in terms of diagnostic strategy [6, 9–14], and optimal oxygenation and ventilation strategy [13–15]. Studies have reported the need for prompt identification of the ARF etiology, as this remains a major determinant of mortality [16].

Viral pathogens are increasingly detected in both immunocompetent and immunocompromised patients with acute respiratory failure [17]. In addition to climatic challenges, high-dose therapies and aggressive treatments to control underlying diseases might be at stake. Furthermore, the development of molecular tools such as multiplex PCR assays over the past 10 years might have shed light on previously undocumented pneumonia in this setting. According to three recently published meta-analyses investigating the incidence of respiratory virus infection in immunocompetent adult patients with community-acquired pneumonia, the pooled proportion of virus pneumonia ranged from 22% to 24.5% [18–20]. The incidence is less precisely known in immunocompromised patients. A recent study has suggested that a virus was detected in 21.3% of 747 cancer patients admitted to ICU for various reason [21]. Moreover, in this study, virus detection in upper airways was independently associated with mortality [21]. However, outcomes associated with virus-positive acute respiratory failure (virus-detected ARF) in immunocompromised patients remain unclear and data are needed to address this specific clinical question.

In the present study, we aimed to describe the spectrum of critically ill immunocompromised patients with virus-associated pneumonia and to report outcomes of virus-detected ARF. We also compared the survival of patients with virus-detected ARF to those admitted to the ICU for ARF due to other etiologies.

## Methods

### Population and study design

Data reported in Table 1 were prospectively collected. Noted that some data have been previously published [4, 13, 14, 22–25]. The study was performed using the database from a multicentric collaborative group specialized in the management of immunocompromised patients, the *Groupe de Recherche en Réanimation Respiratoire en Onco-Hématologie* (GRRR-OH). Briefly, this cohort included data from more than 4000 immunocompromised patients with ARF from 72 ICUs in France. The inclusion period ranged from 2003 to 2017. All management decisions were made independently at each center according to standard practices. In each center, patients underwent a global comprehensive assessment to identify ARF etiologies, which was either invasive (e.g., fiberoptic bronchoscopy with bronchoalveolar lavage, FO-BAL) and/or noninvasive. Noninvasive tools included: blood and sputa cultures, serology, serum and urine antigens, PCR in blood, serum and nasopharyngeal aspirates, high-resolution CT scan, and echocardiography. Details about mortality and diagnosis strategy variations across centers are given in Additional file 1: Table S1.

**Table 1:** Main baseline characteristics and clinical outcomes according to hospital status in immunocompromised patients with virus detected acute respiratory failure

Variable	Overall *N* = 370 *N* (%) or Median [IQR]	Hospital discharge status		*p* value
		Alive *N* = 230 *N* (%) or Median [IQR]	Dead *N* = 140 *N* (%) or Median [IQR]	
Age, years	63 [52–70]	63 [54–70]	63 [51–70]	0.84
Female gender	144 (39)	90 (39)	54 (39)	1.00
Chronic respiratory disease	61 (17)	44 (20)	17 (13)	0.08
Tobacco use	66 (23)	47 (26)	19 (17)	0.08
Charlson score	4 [2–6]	4 [3–6]	4 [3–6]	0.53
PS score ≥ 2	141 (43)	79 (39)	62 (51)	0.04
Underlying conditions				
Immunosuppression category				0.33
Hematological malignancy	234 (63)	141 (61)	93 (66)	
Acute leukemia	69 (19)	33 (14)	36 (26)	
Lymphoma	82 (22)	53 (23)	29 (21)	
Multiple myeloma	68 (18)	49 (21)	19 (14)	
Others	15 (4)	6 (3)	9 (6)	
Solid tumor	52 (23)	31 (13)	21 (15)	
Other	84 (14)	58 (25)	26 (19)	
Solid organ transplant	40 (11)	27 (12)	13 (9)	
Drugs	44 (12)	31 (13)	13 (9)	
Allogeneic–HCT	57 (15)	33 (14)	24 (17)	0.57
Valacyclovir prophylaxis*	90 (35)	48 (31)	42 (40)	0.16
Clinical characteristics at ICU admission				
Neutropenia	146 (40)	84 (37)	62 (46)	0.09
Platelet count (10^9/L)	104 [35–188]	122 [54–199]	62.5 [27.5–150]	< 0.001
PaO_2_/FiO_2_ on day 1, mmHg	126 [91–173]	130 [95–173]	116 [83.5–169]	0.08
≥ 2 involved quadrants on chest X-ray	266 (86)	163 (86)	103 (87)	0.98
Oxygenation strategy at day 1				
NIV	105 (28)	64 (28)	41 (29)	0.81
High-flow oxygen	116 (31)	63 (27)	53 (38)	0.04
Invasive mechanical ventilation	95 (26)	47 (20)	48 (35)	0.003
SOFA score	6 [4–9]	5 [4–8]	8 [5–11]	< 0.001
Detected virus				
Influenza	219 (56)	135 (58)	84 (60)	0.09
Influenza-like	95 (24)	69 (29)	26 (19)	
Respiratory Syncytial virus	54	40	14	
Parainfluenza virus III	23	14	9	
Human Metapneumovirus	18	15	3	
Others	59 (15)	45 (20)	31 (22)	
Rhinovirus	22	11	11	
Adenovirus	11	8	3	
Coronavirus	8	4	4	
Others**	18	22	13	
Coinfection viral–viral***	18 (5)	14 (6)	4 (3)	0.25
ICU stay				
Invasive mechanical ventilation (overall)	141 (38)	65 (28)	76 (54)	< 0.001
Vasopressor support	202 (55)	90 (39)	112 (80)	< 0.001
RRT	64 (17)	28 (12)	36 (26)	0.001
Oseltamivir	98 (30)	52 (16)	46 (14)	0.11
Steroids	112 (34)	78 (21)	49 (13)	0.51
Outcomes				
Treatment withdrawal	103 (28)	12 (5)	91 (65)	< 0.001
Invasive mechanical ventilation duration, days	9 [4–18.6]	10 [4–18]	9 [3–19]	0.51
ICU length of stay, days	8 [4–17]	6 [4–15]	10 [5–20]	0.003
Hospital length of stay, days	17 [11–34]	19 [12–34]	15 [10–32]	0.19
In-ICU mortality	113 (31)	–	–	–
In-hospital mortality	140 (38)	–	–	–

^*^Missing data in 112 patients

^**^Others = Enterovirus (*n* = 3); HHV6 (*n* = 2); HSV (*n* = 9); Others (*n* = 4)

^***^Influenza/RSV (*n* = 3); Rhinovirus/Enterovirus (*n* = 2); Influenza/Rhinovirus/Enterovirus (*n* = 2); Influenza/HSV (*n* = 1); Influenza/PIV3 (*n* = 1); Influenza/Rhinovirus (*n* = 1); Human Metapneumovirus/Rhinovirus/Enterovirus (*n* = 1); PIV3/Coronavirus (*n* = 1); PIV3/Human Metapneumovirus (*n* = 1); PIV3/RSV (*n* = 1); Rhinovirus/Human Metapneumovirus (*n* = 1); RSV/Coronavirus (*n* = 1); RSV/Human Metapneumovirus (*n* = 1); RSV/Rhinovirus (*n* = 1)

Allogeneic–HCT: allogeneic hematopoietic stem-cell transplantation; NIV: non-invasive ventilation; PS score: Performance Status score; RRT: renal replacement therapy; SOT: solid organ transplant; SOFA score: Sepsis Organ Failure Assessment score;

For each patient, four investigators (EA, VL, AK, and DM) analyzed the charts blinded from the diagnosis established by the clinicians in charge. Neutropenia was defined on ICU admission as an absolute neutrophil count < 1000/mm^3^. Invasive fungal infections were defined according to the European Organization of Research and Treatment of Cancer/Mycosis Study Group (EORTC/MSG) group guidelines [26]. Only probable or proven aspergillosis have been taken into account according to host factors and clinical features (Chest CT aspect, bronchoscopy aspect, results from galactomannan antigen (in serum and/or bronchoalveolar lavage) or Aspergillus PCR). Bacterial pneumonia was defined as clinically or microbiologically documented low respiratory tract infection.

The main objective was to investigate the frequency and severity of acute respiratory failure from a viral origin in immunocompromised patients compared to ARF from other origins. We also sought to identify factors associated with in-hospital mortality. To do so, we first identified patients with acute hypoxemic respiratory failure by applying the following inclusion criteria: adult patients (≥ 18 years) with hypoxemic ARF (PaO_2_ < 60 mmHg and/or SpO_2_ < 90% on room air and/or tachypnea > 30/min and/or signs of respiratory distress, such as labored breathing, and/or the need for more than 6L/min oxygen), admitted to the ICU with non-Acquired ImmunoDeficiency Syndrome underlying immunosuppression: hematologic malignancy or solid tumor (active or treated for less than 5 years), hematopoietic stem cell transplants, solid organ transplantation, high dose (> 0.5 mg/kg/day) or prolonged (> 3 months) steroids or other immunosuppressive drugs. Exclusion criteria were ARF related to acute pulmonary edema and ARF of unknown origin (e.g., without a definite diagnosis).

Patients with a diagnosis of virus-detected ARF were identified and we investigated their characteristics as well as factors associated with mortality. We then performed a case–control study to assess survival in virus-detected ARF (cases) as compared to ARF from other causes (controls). Viruses were split into three groups: influenza virus, influenza-like viruses, and others. Influenza-like viruses included respiratory syncytial virus (RSV), parainfluenza virus (PIV), and human metapneumovirus (hMPV) which share a common phylogenetic family (the paramyxoviridae) and similar clinical tropism.

### Statistical analysis

Continuous variables are described as median and interquartile range (IQR) or mean (± SD) and compared using Wilcoxon’s rank sum test; categorical variables are shown as counts (percent) and compared using Fisher’s exact test.

The main outcome was in-hospital mortality, analyzed as a binary variable. First, to investigate factors independently associated with hospital mortality, we used multivariable logistic regression. To take into account center variations, mixed-effect models were used with the center as a random variable. The model was built using a conditional backward stepwise variable selection process based upon variable influence in univariate analysis. Critical entry and exit *p* values were 0.2 and 0.1, respectively. It was preplanned to force clinically relevant variables (type of virus) into the final model if they were not previously selected. Log-linearity assumption was checked, and variables were tested for collinearity before inclusion in the multivariable model. The goodness-of-fit was evaluated using the le Cessie–van Houwelingen test and discrimination with C-statistic. The final model was assessed by calibration, discrimination, and relevance.

Thereafter, for the case–control analysis, a matching procedure was performed. Patients with virus-detected ARF were individually matched in a 1:3 ratio to a control group of immunocompromised patients with ARF of other causes, without replacement. The matching criteria were: age (exact match), year of ICU admission (exact match), PaO2/FiO2 (0.1 SD), SOFA score (exact match), underlying immunosuppression (exact match), and neutropenia status (exact match). Balances in patients’ characteristics before and after matching were assessed using standardized mean differences. We used generalized estimating equations stratified on clusters to compare in-hospital mortality according to ARF causes. All analyses were performed on complete cases.

The measures of associations are presented with odds ratios and confidence intervals at 95%. All tests were two-sided and *p* values lower than 5% were considered to indicate significant associations. Analyses were performed using R statistical platform, version 3.0.2 (https://cran.r-project.org/).

## Results

### Characteristics of immunocompromised patients with virus-detected ARF

**Fig. 1:**
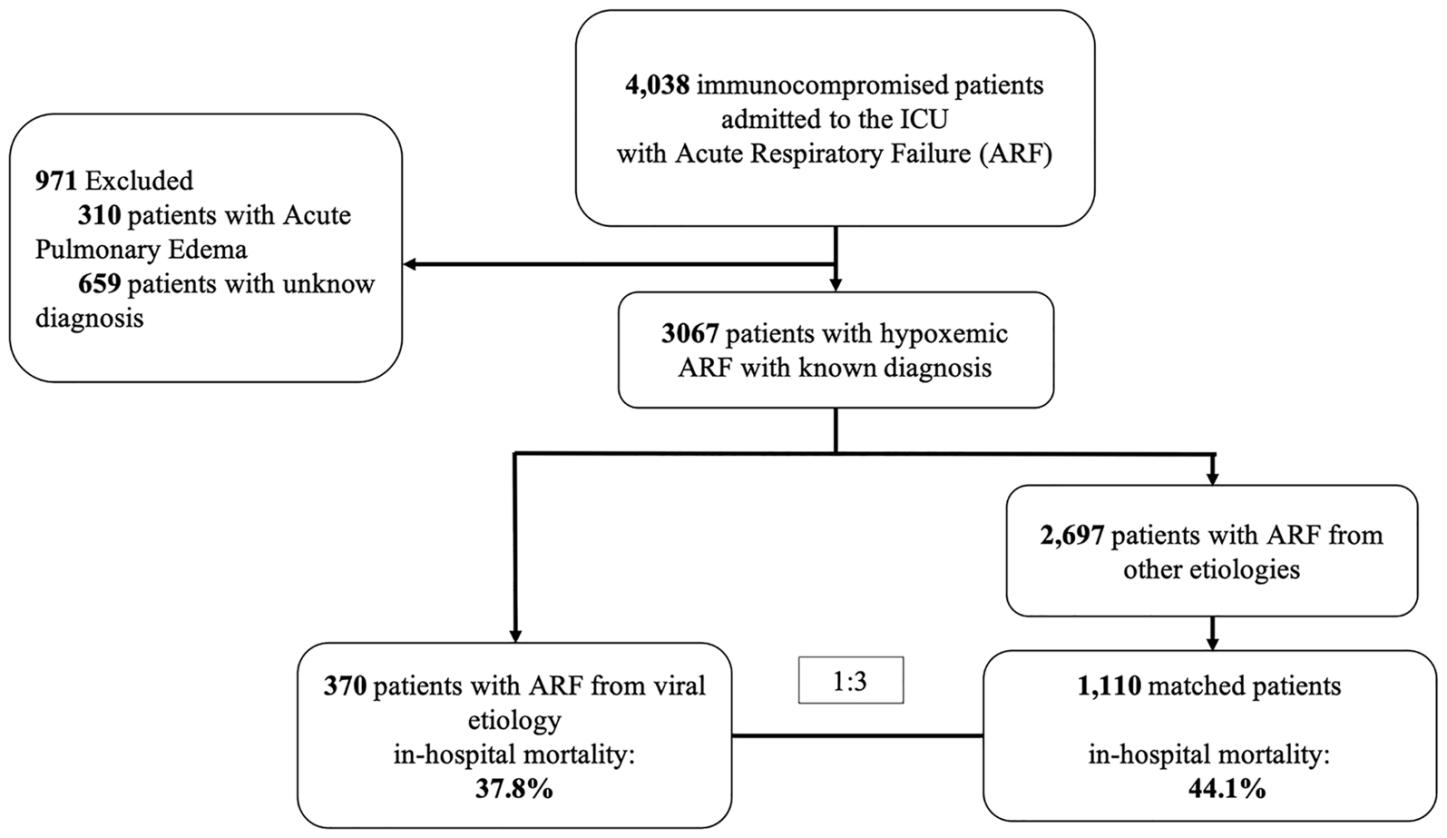
Flow chart of the study

Of the 4038 critically ill patients with ARF, 370 (9.2%) had a confirmed diagnosis of viral infection (Fig. 1 and, Additional file 1: Fig. S2.

Two-hundred and twenty-three patients (61%) were male and the median age was 63 [52–70] years. Overall, 234 (63%) patients had a hematological malignancy, mainly lymphoproliferative disorders (Table 1) and 57 (15%) had received an allogeneic hematopoietic stem cell transplant. Vaccination coverage was low: 30 patients have received seasonal influenza vaccine (16%, 181 missing data) and 20 patients pneumococcal vaccination (10%, missing values: 44%).

At admission, the median SOFA score was 6 [4–9] and the median PaO_2_/FiO_2_ ratio was 126 [91–173] mmHg. Ninety-five (26%) patients needed first-line invasive mechanical ventilation, 105 (28%) non-invasive ventilation, and 116 (31%) high-flow nasal oxygen therapy. Throughout the ICU stay, 141 (38%) patients required mechanical ventilation with a median duration of ventilation of 9 [4–19] days.

Regarding infection management, 98 patients have received Oseltamivir (26.4%) through ICU stay, 112 (30%) steroids, and all patients have been treated with antibiotics for at least 2 days.

The crude ICU and Hospital mortality rates were 31% and 38%, respectively (Table 1).

Additional file 1: Table S2 depicts temporal changes in first-line oxygenation strategy choice and mortality across years. As shown, there was an increasing use of high-flow nasal cannula oxygen and mortality significantly decreased over time (*p* < 0.01).

### Characteristic isolated viruses

Overall, 388 viruses have been identified in 370 patients (Table 1). The investigational procedure performed to establish the diagnosis is summarized in Additional file 1: Table S1. As shown, viruses have been mainly identified in a nasopharyngeal swab (*n* = 268, 72%), followed by bronchoalveolar lavage (*n* = 187; 50%) and other non-protected respiratory samples (*n* = 117; 32%). Sixty-three percent of the patients had the same pathogen identified in both the upper and lower tract samples.

Influenza was the most frequently identified virus (58%, *n* = 227), followed by RSV (15%, *n* = 61), and parainfluenza virus III (6%, *n* = 26). Eighteen patients have more than one identified virus in their respiratory sample (viral–viral coinfections, Table 1).

We found a seasonal trend with 220 (59%) infections in winter compared to 89 (24%) in autumn, 48 (13%), and 13 (4%) in spring and summer, respectively (*p* < 0.01).

The virus distribution according to immunosuppression is displayed in Additional file 1: Fig. S3. While influenza-like viruses and other viruses were found in similar proportions for each type of underlying immunosuppression, influenza virus was particularly prevalent in patients with hematological malignancies other than acute myeloid leukemia and allograft.

**Fig. 2:**
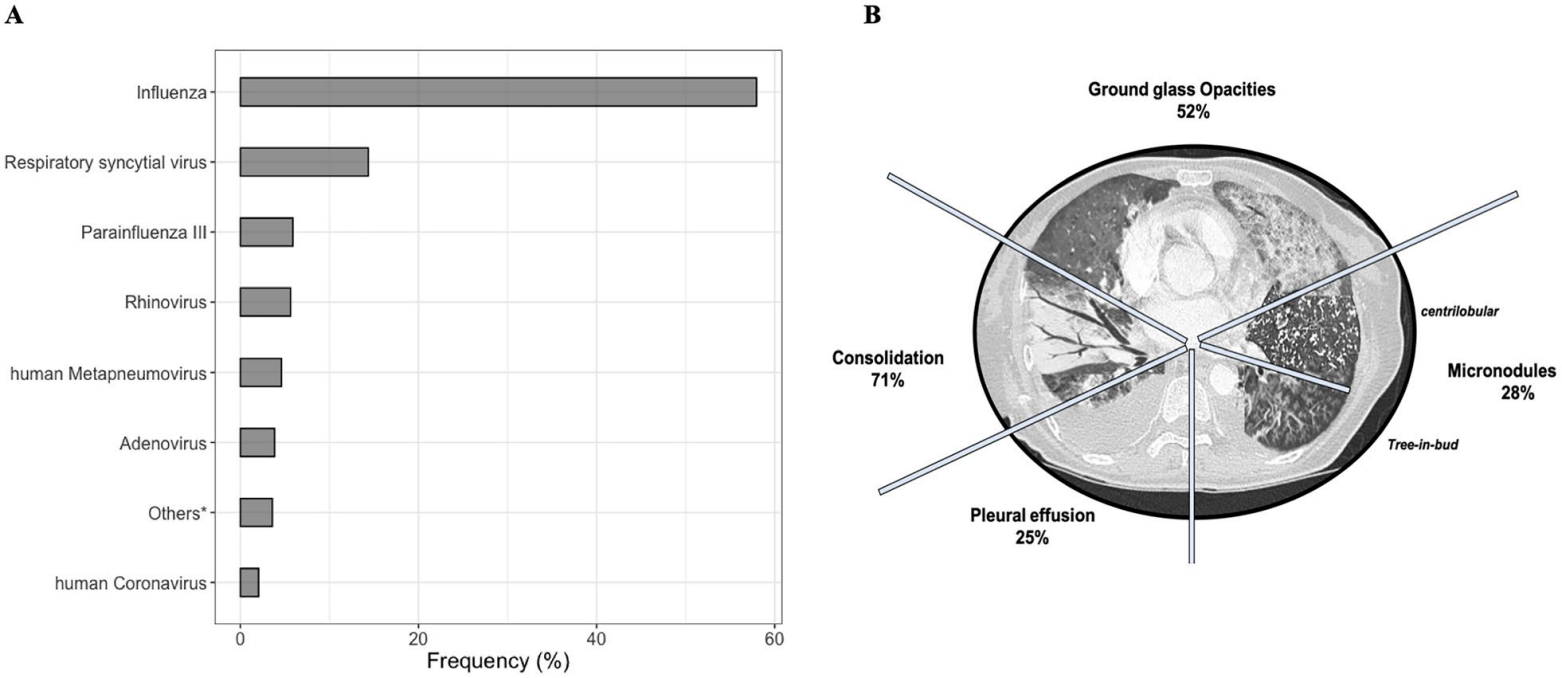
Distribution of virus species in a cohort of 370 patients (**A**). Main radiological patterns identified on 189 chest CT-scan in patients with virus-associated pneumonia (**B**). * Others: enterovirus, rhinovirus, human coronavirus, HHV6, HSV

Chest X-ray usually demonstrated diffuse lung infiltration with an interstitial pattern (89%, Table 1), while bilateral ground glass opacities (52%, *n* = 106) and pulmonary nodules (28%, *n* = 57) were the most frequent lesions encountered on the chest-CT scan (Fig. 2 and Additional file 1: Table S2).

Patients with RSV and influenza infection shared a very similar clinical presentation, except for a higher frequency of running noses and less severe hypoxemia among the first (Additional file 1: Table S3).

Overall, an associated bacterial infection was identified in 79 patients (21%, 50 patients with influenza infection, 17 with influenza-like viruses, 12 with others viruses), and invasive pulmonary aspergillosis in 23 patients (6%, 15 patients with influenza infection, 3 with influenza-like viruses, and 5 with others viruses). Cocci Gram-positive pathogens were the most commonly identified (56%), mainly Streptococcus pneumoniae (*n* = 27, 34%) and Staphylococcus aureus (*n* = 14, 18%). Details about co-infection pathogens and invasive pulmonary aspergillosis diagnosis are given in Additional file 1: Tables S4 and S5.

Crude mortality rate according to virus species is displayed in Additional file 1: Fig. S4.

### Outcomes of immunocompromised patients with virus-detected ARF

Factors associated with in-hospital mortality in univariate analysis are described in Table 1. By multivariable analysis, independent factors associated with hospital mortality were: poor performance status (OR = 1.84 [1.12–3.03]), neutropenia at ICU admission (OR: 1.74 [1.05–2.89]), and the need for endotracheal intubation on the day of admission (OR = 1.97 [1.14–3.40]). We did not find any significant association between the type of detected virus and mortality (Table 2).

**Table 2:** Factors associated with in-hospital mortality in 370 critically ill immunocompromised patients with virus-detected respiratory failure

	OR [95% CI]	*p* value
Detected virus		0.09
Other respiratory viruses	Reference level	
Influenza	1.03 [0.53–2.04]	
Influenza-like*	0.51 [0.22–1.17]	
Neutropenia at admission	1.74 [1.05–2.89]	0.03
Invasive mechanical ventilation, day of admission	1.97 [1.14–3.40]	< 0.01
Performance status ≥ 2	1.84 [1.12–3.03]	0.01

Multivariate mixed effects model with random effect on center (c-index = 0.73; *p* value Hosmer–Lemeshow = 0.898)

^*^Parainfluenza virus, human metapneumovirus, and respiratory syncytial virus

In addition, neither associated invasive pulmonary aspergillosis (adjusted OR = 1.96 [0.64–6.00]) nor bacterial infection (adjusted OR = 1.24 [0.58–2.66]) were associated with mortality, as well as viral–viral coinfections (adjusted OR = 0.58 [0.18–1.88]). As the same, Oseltamivir (adjusted OR = 1.53 [0.88–2.67]) and steroids used (adjusted OR = 1.12 [0.67–1.85]) were not associated with hospital mortality.

### Matched comparison of critically ill immunocompromised patients with virus-detected ARF and a control group with ARF from other etiologies

All patients with virus-detected ARF were matched with 1100 patients with ARF of other causes (Fig. 1). As shown in Table 3 and Additional file 1: Fig. S5, cases and controls were well-matched. The main cause of ARF in the control group was bacterial infection (*n* = 637, 58%) followed by tumor-related ARF (*n* = 258, 23.5%) and pneumocystis pneumonia (*n* = 120, 10.8%).

**Table 3:** Description of patients before and after matching with patients with respiratory failure from other etiologies

	Before matching			After matching	
	Viral–ARF *N* = 370	Other ARF *N* = 2697	Absolute standardized mean difference	Other ARF *N* = 1110	Absolute standardized mean difference
Age, years	60 (14)	60 (15)	0.004	60 (15)	0.014
Female gender	147 (39)	1018 (38)	0.036	418 (38)	0.037
Chronic respiratory disease	61 (17)	604 (24)	0.176	235 (23)	0.135
Tobacco use	66 (23)	642 (33)	0.222	241 (32)	0.214
Charlson score	4 (2)	5 (3)	0.319	5 (3)	0.209
PS score ≥ 2	141 (43)	1124 (49)	0.119	492 (50)	0.138
Underlying conditions					
Immunosuppression category			0.465		0.039
Hematological malignancy	234 (63)	1589 (59)		673 (60)	
Acute leukemia	69 (19)	705 (26)		224 (20)	
Lymphoma	82 (22)	565 (21)		269 (24)	
Multiple myeloma	68 (18)	181 (7)		107 (9)	
Others	15 (4)	138 (5)		73 (6)	
Solid tumor	52 (23)	685 (25)		261(23)	
Other	84 (14)	423 (16)		176 (16)	
Solid organ transplant	40 (11)	226 (8)		105 (14)	
Drugs	44 (12)	685 (7)		156 (9)	
Allogeneic–HCT	57 (15)	265 (10)		161 (15)	
Neutropenia	146 (40)	820 (30)	0.191	407 (37)	0.058
ICU admission					
Invasive mechanical ventilation	95 (26)	714 (27)	0.027	268 (24.5)	0.028
NIV	105 (28)	658 (25)	0.089	258 (23)	0.116
High-flow oxygen	116 (31)	643 (24)	0.169	369 (33)	0.040
PaO_2_/FiO_2_, mmHg	141 (67)	165 (91)	0.302	140 (66)	0.013
SOFA score	7 (4)	7 (4)	0.032	7 (4)	0.002
ICU stay					
Invasive mechanical ventilation (overall)	141 (38)	1239 (46)	0.160	488 (44)	0.120
Vasopressor support	202 (55)	1454 (54)	0.013	597 (54)	0.014
RRT	64 (17)	522 (19)	0.055	189 (17)	0.004
Outcomes					
Treatment withdrawal	103 (28)	738 (27)	0.009	317 (29)	0.018
Invasive mechanical ventilation duration, days	14 (14)	8 (14)	0.076	13 (17)	0.021
ICU length of stay, days	15 (26)	11 (15)	0.195	11 (13)	0.215
Hospital length of stay, days	24 (16.53)	27 (25)	0.129	29 (28)	0.238
In ICU Mortality	113 (31)	839 (31)	0.012	359 (32)	0.039
In hospital Mortality	140 (38)	1161 (43)	0.106	490 (44)	0.128

Results are presented as *N* (%) and mean (SD). Absolute standardized mean difference (SMD) is the absolute value of the difference in mean between groups divided by standard deviation. An absolute standardized mean difference of less than 0.2 usually shows a balance between groups

Allogeneic–HCT: allogeneic hematopoietic stem-cell transplantation; ARF: acute respiratory failure; ICU: intensive care unit; NIV: non-invasive ventilation; PS score: Performance Status score; RRT: renal replacement therapy; SMD: Standardized Mean Differences; SOFA score: Sepsis Organ Failure Assessment score; SOT: solid organ transplant

**Fig. 3:**
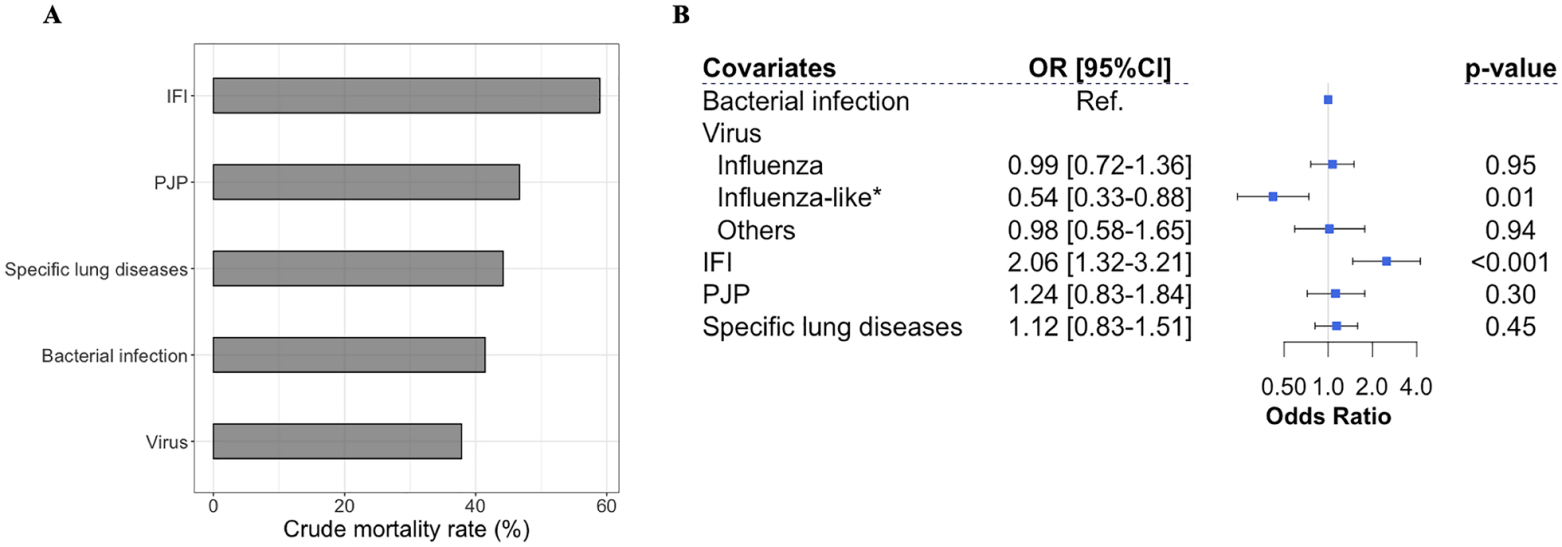
Primary outcomes on the matched cohort. Crude hospital mortality according to diagnosis category (**A**). Pair matched odds ratio according to diagnosis category (**B**). PJP: Pneumocystis Jirovecii Pneumonia; IFI: Invasive Fungal Infection (*N* = 114; Invasive Pulmonary Aspergillosis *n* = 72, Candida sp. *n* = 16, Fusarium sp. *n* = 3, Trichosporum sp. *n* = 3, Mucormycosis *n* = 1). * Others: enterovirus, rhinovirus, human coronavirus, HHV6, HSV

After matching, the overall in-hospital mortality across patients with or without virus-detected ARF was 37.8% (*n* = 140) and 44.1% (*n* = 490), respectively (*p* = 0.004) (Fig. 3A).

Patients with virus-detected ARF had significantly lower mortality than ARF from other etiologies (OR = 0.77 [0.60–0.98], *p* = 0.03).

We then considered each diagnosis separately, with bacterial infection as a reference. As shown in Fig. 3B, compared to bacterial pneumonia, influenza-like viruses identification was significantly associated with a better outcome (OR = 0.55 [0.34–0.89], *p* = 0.02), while influenza infection (OR 0.99 [0.72–1.36], *p* = 0.95) and other viruses (OR 0.97 [0.57–1.65], *p* = 0.91) did not reach statistical significance. Of note, invasive fungal infection was significantly associated with mortality (OR = 2.06 [1.32–3.21], *p* < 0.001). These results did not change by taking into account viral–bacterial or viral–aspergillosis co-infection groups or the exclusion of patients with bacterial pneumonia (Additional file 1: Tables S6 and S7).

## Discussion

In this large cohort of critically ill immunocompromised patients with ARF, we found that virus-detected ARF was a common reason for ICU admission, especially during winter and fall times. Influenza was the leading virus. In-hospital mortality remains high, mainly driven by ARF severity and associated organ dysfunctions, especially in patients with altered health status. Interestingly, mortality did not vary across the type of virus, even though patients with virus-detected ARF had a higher survival rate than those with ARF from other etiologies.

Since the development of routine molecular testing, in particular multiplex PCR assay, there is growing attention to virus-detected pneumonia [21, 27, 28]. However, data are scarce in immunocompromised patients. In this study, influenza virus was the most frequently identified virus with crude mortality near 40% in this population. Interestingly, we found a significant variation in the type of virus identified according to the immunosuppression underlying. This might be explained by differences in seroconversion and/or seroprotection within the different types of immunosuppression, especially for lymphoproliferative diseases and solid organ transplantation [29, 30]. For example, it has been found a dose-dependent correlation between mycophenolate mofetil use and frequency of seroconversion after influenza vaccine [31]. Along this line, in a meta-analysis conducted in 1966 patients with systemic lupus erythematous, seroprotection rate was significantly low compared to general population [32].

Although viruses were generally the sole infectious agents identified, we found frequent bacterial coinfection. This highlights the need to discuss prompt antibiotic therapy whatever the type of immunosuppression [3, 33], and even more so in the case of associated neutropenia [3, 34]. In this line, we found a high rate of associated invasive aspergillosis, and vigilance should be maintained in case of viral infection [35, 36], especially in patients with other risk factors (e.g., neutropenia, steroids, hematological malignancies) [37, 38].

The overall mortality remains high, but in accordance with previous studies [13, 14]. We did not found significant variation of mortality according to the underlying immunosuppression, although acute myeloid leukemia has been previously associated with mortality excess [39]. The prognosis was mainly related to the severity of the disease, two factors already reported in the literature [4, 16, 21, 34]. The prognostic impact of neutropenia is debatable overall, and especially in viral pneumonia, where it was not associated with mortality in a large cohort of 1481 critically ill immunocompromised patients admitted to the ICU for ARF [40]. Its presence may reflect a particular type of immunosuppression with an increased risk of invasive pulmonary aspergillosis, which has an appalling prognosis with up to 75% mortality at 90 days [38]. In patients with virus-detected ARF, we did not find any association between the type of virus identified and mortality. However, compared with a control cohort of ARF from other etiologies, this study found a significantly lower mortality rate in patients with virus-detected pneumonia (38% compared to 44%), especially with influenza-like viruses. This result is in line with a previous study of 604 immunocompromised patients with ARF [16], in which invasive pulmonary aspergillosis and ARF without definite diagnosis were associated with mortality contrary to viral infection. These contrasting findings may be explained by the limited therapeutics option in some ARF etiologies on one hand and the relevance of virus detection in such patients on the other hand. Indeed, the pathogenicity of some viruses (especially influenza-like or rhinovirus) may be difficult to assess especially when viruses are detected in the upper respiratory tract. Interestingly, Legoff et al., have shown that virus detection in the upper airway (whatever the type) was associated with increased ICU mortality, even in patients without respiratory symptoms [21]. In addition, mortality rates from respiratory virus infections are quite high in immunocompromised patients, ranging from 21% to 83% in cases of RSV infection [41] and 27% in hMPV [42] and PIV [43]. This suggests that viruses can not only play the role of a bystander but also lead to severe infections or trigger another respiratory event (such as organized pneumonia, for example).

This study has several limitations. First, because of the retrospective design, unidentified confounding factors may have been overlooked. Second, there were no standardized guidelines for the method used to identify viral pathogens (upper and/or lower respiratory tract, blood sample), and the panel used for virus detection has varied over the years and across centers, which could have introduced some heterogeneity and underestimated virus-detected ARF frequency. To reduce a potential bias in our results, a panel of 4 experts reviewed all the diagnoses and procedures, and only patients with a definite diagnosis were included. In addition, we used the year of ICU admission in the matching process to allow comparisons of patients admitted during the same time period, and the center effect has been taken into account. Nevertheless, we cannot rule out some residual uncertainty in our findings. As the same, the study design did not allow us to identify the precise link between virus exposure and mortality, in particular for viruses other than Influenza and those detected in the upper respiratory tract only. Future studies are warranted to answer the precise clinical significance of virus detection as the correlation between the underlying immunosuppression and host susceptibility. Fourth, the large study period may have influenced virus incidence and prognosis according to underlying malignancy, due to therapeutic advances and new mechanisms of effect. Finally, most of the participating centers are tertiary centers with important expertise in the management of immunocompromised patients which could limit the generalizability of our findings.

In conclusion, from a large cohort of immunocompromised patients, we found a high mortality rate associated with virus-detected respiratory failure but lower than other causes of ARF in this setting, in particular for influenza-like viruses. Clinical severity at ICU admission, neutropenia as well as patient general status are the main determinants of mortality. We did not find any protective factors suggesting the importance of preventive strategies in this high risk population.

### Supplementary Information

**Additional file 1.** **Figure S1.** Center effect study on hospital mortality rate (panel A), bronchoalveolar lavage (panel B), and nasopharyngeal aspiration (panel C) procedure; **Figure S2.** Temporal changes in mortality (Panel A), first-line oxygenation/ventilation strategy (Panel B), and virus detection (Panel C) across years; **Figure S3.** Distribution of virus species according to immunosuppression; **Figure S4.** Crude mortality rate according to virus species; **Figure S5.** Absolute standardized mean difference between patients with and without virus-associated acute respiratory failure, before and after matching. **Table S1.** Overview of investigational procedures performed in the whole cohort and the respiratory-virus cohort; **Table S2.** Description of radiological pattern in critically-ill patients with virus-associated acute respiratory failure; **Table S3.** Clinical characteristics and outcomes comparisons across patients with respiratory syncytial virus or Influenza infection; **Table S4.** Description of documented bacterial co-infections; **Table S5.** Clinical characteristics, risk factors, and results from investigational procedures in 23 patients with virus-detected respiratory failure and documented invasive pulmonary aspergillosis; **Table S6.** Pair matched odds ratio for hospital mortality according to diagnosis category, taking into account co-infections; **Table S7.** Pair matched odds ratio for hospital mortality according to diagnosis category after excluding patients with bacterial pneumonia; **Table S8.** Factors associated with in-hospital mortality in 370 critically ill immunocompromised patients with virus-detected acute respiratory failure taking into account co-infections

## Data Availability

The data sets used and/or analyzed during the current study are available from the corresponding author upon reasonable request.

## Abbreviations

AIDS: Acquired immunodeficiency syndrome
ARF: Acute respiratory failure
FO-BAL: Fiberoptic bronchoscopy with bronchoalveolar lavage
ICU: Intensive care unit
IQR: Interquartile range
hMPV: Human Metapneumovirus
OR: Odds ratio
PIV: Parainfluenza virus
RSV: Respiratory syncytial virus
SOFA: Sequential organs failure assessment score
