# Supplementary material for: Prognosis of critically ill immunocompromised patients with virus-detected acute respiratory failure
Source: Ann Intensive Care. 2023 Oct 13;13:101. doi: 10.1186/s13613-023-01196-9 (PMC10575827; doi:10.1186/s13613-023-01196-9)
Supplement: Supplementary file 1 — Additional file 1. Figure S1. Center effect study on hospital mortality rate (panel A), bronchoalveolar lavage (panel B), and nasopharyngeal aspiration (panel C) procedure; Figure S2. Temporal changes in mortality (Panel A), first-line oxygenation/ventilation strategy (Panel B), and virus detection (Panel C) across years; Figure S3. Distribution of virus species according to immunosuppression; Figure S4. Crude mortality rate according to virus species; Figure S5. Absolute standardized mean difference between patients with and without virus-associated acute respiratory failure, before and after matching. Table S1. Overview of investigational procedures performed in the whole cohort and the respiratory-virus cohort; Table S2. Description of radiological pattern in critically-ill patients with virus-associated acute respiratory failure; Table S3. Clinical characteristics and outcomes comparisons across patients with respiratory syncytial virus or Influenza infection; Table S4. Description of documented bacterial co-infections; Table S5. Clinical characteristics, risk factors, and results from investigational procedures in 23 patients with virus-detected respiratory failure and documented invasive pulmonary aspergillosis; Table S6. Pair matched odds ratio for hospital mortality according to diagnosis category, taking into account co-infections; Table S7. Pair matched odds ratio for hospital mortality according to diagnosis category after excluding patients with bacterial pneumonia; Table S8. Factors associated with in-hospital mortality in 370 critically ill immunocompromised patients with virus-detected acute respiratory failure taking into account co-infections [file 13613_2023_1196_MOESM1_ESM.docx]

# **Prognosis of Critically-ill Immunocompromised patients with Virus-detected Acute Respiratory Failure**

Guillaume Dumas, Maxime Bertrand, Virginie Lemiale, Emmanuel Canet, François Barbier, Achille Kouatchet, Alexandre Demoule, Kada Klouche, Anne-Sophie Moreau, Laurent Argaud, Florent Wallet, Jean-Herlé Raphalen, Djamel Mokkart, Fabrice Bruneel, Frédéric Pène, Elie Azoulay

**Supplementary data**

1-Study of the center effect on hospital mortality and procedural investigations………..…p2

2-Characteristics of immunocompromised patients with virus-detected ARF……………...p3

3-Matched comparison of critically-ill immunocompromised patients with viral-ARF

and a control group with ARF from other etiologies……………………………………......p9

4-Sensitivity analyses ……………………………………………………………………….p10

5-References…………………………………………………………………………………p12

**1-Study of the center effect on hospital mortality and procedural investigations**

Due to the large number of participating centers, we investigated for a potential center effect in our cohort. We used mixed-effect logistic regression model with center as random variable (random intercept) to examine the variability on outcome between intensive care units. Exchangeability was assumed across all centers. In practice, the effect of a given ICU was modeled through its own regression coefficient, which compares to the crude average outcome across all centers[1, 2]. The significance of the center effect was tested using permutation tests, a recommended approach to test for random effect[3]. The principle is to test whether the random effects variance component is zero. In our situation, the objective is to test if the random effects variance component for center is zero. In practice, we would randomly permute the center that the patient was assigned to, while keeping the number of patients assigned to a given center the same as in the original sample. The null hypothesis is no effect center. Under H0, the outcome is independent of centers, and therefore, the center indices are exchangeable.

The figure below presents these results. As shown, there is no significant center effect on hospital mortality but a significant center effect was detected on BAL and nasopharyngeal swab procedures.


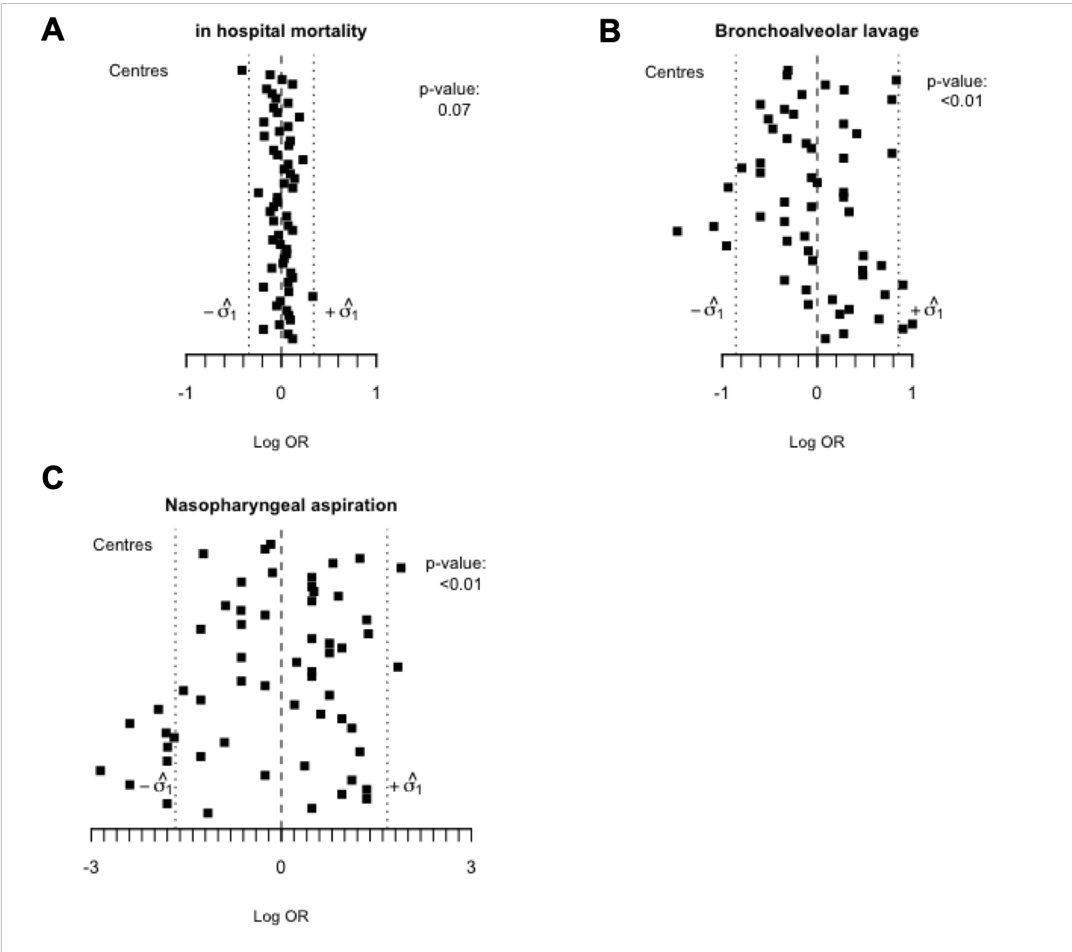
**Figure S1.** Center effect study on hospital mortality rate (panel A), bronchoalveolar lavage (panel B), and nasopharyngeal aspiration (panel C) procedure

**2-Characteristics of immunocompromised patients with virus-detected ARF**

**
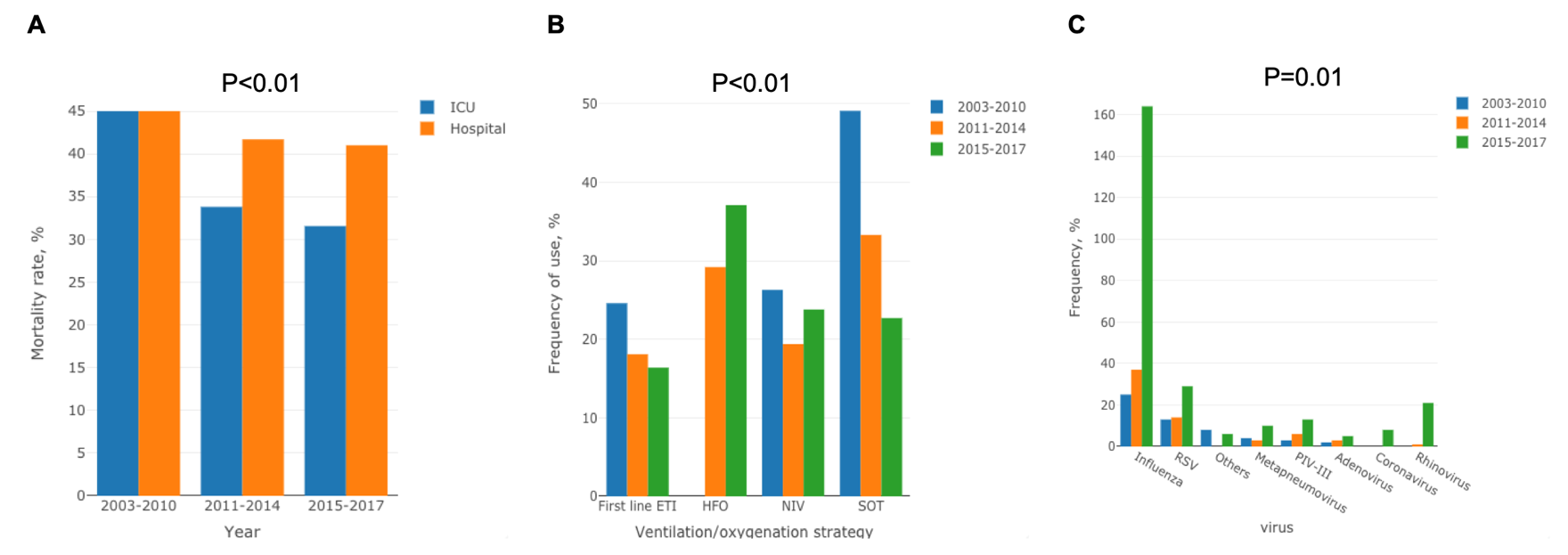
Figure S2.** Temporal changes in mortality (Panel A), first-line oxygenation/ventilation strategy (Panel B), and virus detection (Panel C) across years

*p-value obtained from Cochran-Armitage trend test*


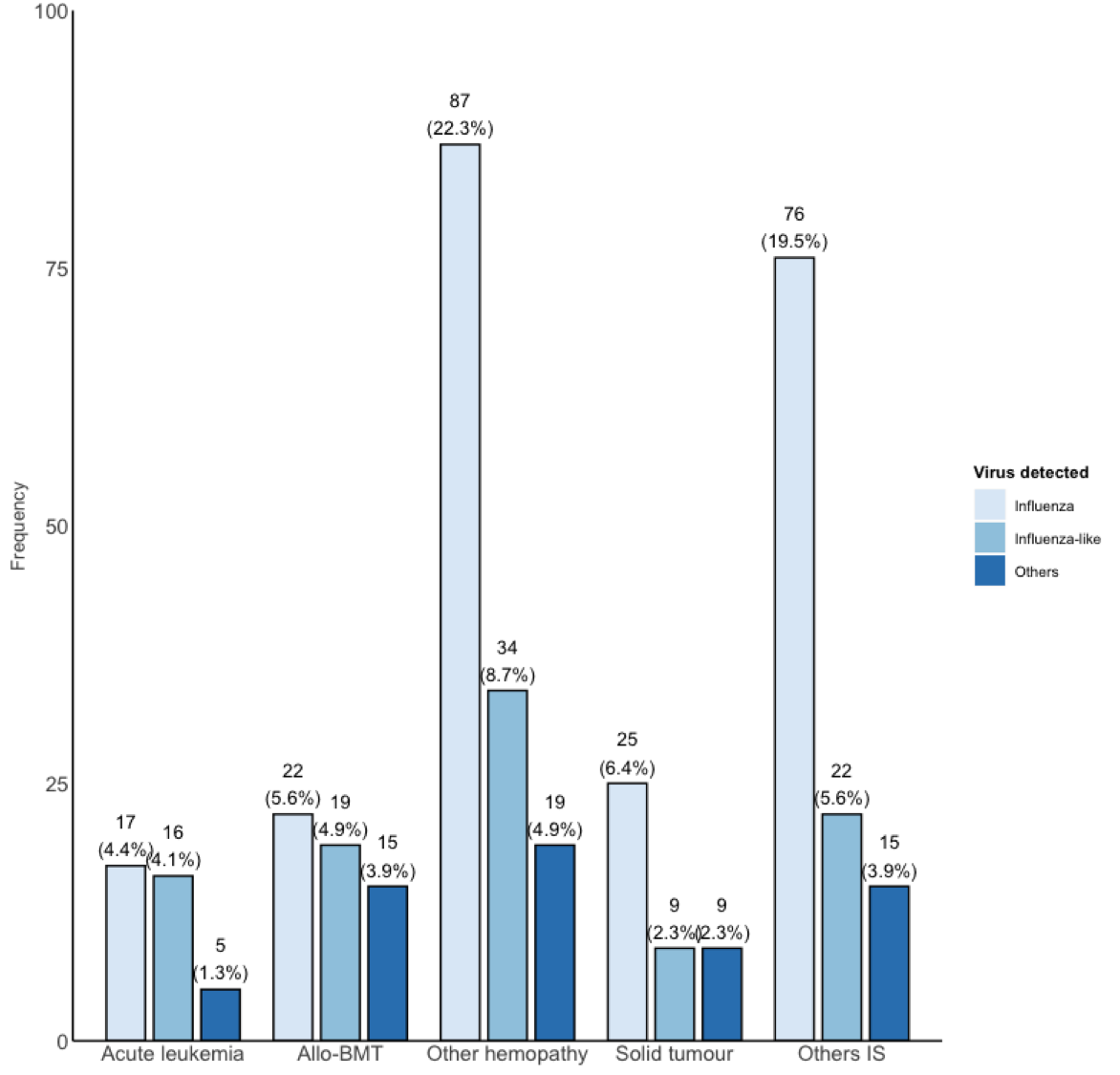
**Figure S3**. Distribution of virus species according to immunosuppression

**Table S1.** Overview of investigational procedures performed in the whole cohort and the respiratory-virus cohort

| **Sample** | **N (%)** |
| --- | --- |
| **Whole cohort** | **4038 (100)** |
| Nasopharyngeal swab | 1503 (37) |
| Fiberoptic bronchoscopy with bronchoalveolar lavage | 1390 (34) |
| Other Non-protected respiratory samples | 3953 (97) |
| **Respiratory-Virus cohort** | **370 (9)** |
| Nasopharyngeal aspirate  Positive samples  Influenza  RSV  Rhinovirus  PIV3  Human Metapneumovirus  Adenovirus  Coronavirus  Enterovirus | 268  262  151  40  20  16  16  8  8  3 |
| Fiberoptic bronchoscopy with bronchoalveolar lavage  Positive samples  Influenza  RSV  PIV3  Rhinovirus  Adenovirus  Human Metapneumovirus  HSV  Coronavirus | 187  174  101  28  13  9  8  7  5  3 |
| Other lower tract respiratory samples  Positive samples  Influenza  RSV  PIV3  Human Metapneumovirus  Rhinovirus  Coronavirus  Adenovirus  Enterovirus  HSV | 117  110  62  14  9  9  5  4  3  3  1 |
| **Samples concordance**:  - Nasopharyngeal aspirate and FO-BAL positive with the same viruses isolated  - Negative Nasopharyngeal aspirate with positive FO-BAL  - Positive Nasopharyngeal aspirate with negative FO-BAL | 91 (70)  37 (26)  15 (11) |

FO-BAL: fiberoptic bronchoalveolar lavage; MPV: human Metapneumovirus; PIV: Parainfluenza virus

RSV: Respiratory Syncytial Virus; HSV: herpes simplex virus.

**Table S2**. Description of radiological pattern in critically-ill patients with virus-associated acute respiratory failure

|  | N (%) |
| --- | --- |
| **Chest X-Ray** | 366 (99) |
| Number of quadrants involved ≥ 2 | 266 (66) |
| Pattern  Focal  Interstitial  Nodular  Pleural | 257 (69)  189 (89)  3 (7)  83 (30) |
| **Chest CT-scan** | 189 (51) |
| Alveolar Lesions  Focal consolidation  Diffuse consolidation  Ground glass opacities  Nodular lesion  Pleural effusion | 76 (38)  59 (29)  106 (52)  57 (28)  80 (25) |

**Table S3.** Clinical characteristics and outcomes comparisons across patients with respiratory syncytial virus or Influenza infection

|  | **Respiratory syncytial virus**  **N=59**  N (%) or Median [IQR] | **Influenza**  **virus**  **N=224**  N (%) or Median [IQR] | **p-value** |
| --- | --- | --- | --- |
| Respiratory sample |  |  |  |
| Nasal swab | 40 (63) | 151 (66) | 1.00 |
| Bronchoalveolar lavage | 28 (45) | 101 (45) | 0.65 |
| Other lower tract respiratory sample | 14 (26) | 60 (29) | 0.38 |
|  |  |  |  |
| Age, years | 60 [54-68] | 63 [52-70] | 0.53 |
| Female gender | 20 (34) | 88 (40) | 0.45 |
| Chronic respiratory disease | 11 (19) | 30 (14) | 0.41 |
| Tobacco use | 21 (43) | 37 (20) | 0.003 |
| Charlson score | 4 [3-7] | 4 [2-6] | 0.57 |
| PS score ≥ 2 | 20 (45) | 85 (45) | 1.00 |
| Immunosuppression category |  |  | 0.26 |
| Hematological malignancy | 33 (58) | 124 (56) |  |
| Acute leukemia | 12 (21) | 31 (14) |  |
| Lymphoma | 11 (19) | 46 (21) |  |
| Multiple myeloma | 8 (14) | 40 (18) |  |
| Others | 2 (4) | 7 (3) |  |
| Solid tumor | 11 (19) | 29 (13) |  |
| Solid organ transplant | 11 (19) | 38 (17) |  |
| Drugs | 2 (4) | 32 (14) |  |
| Allogeneic-HCT | 11 (19) | 23 (10) | 0.11 |
| Season  Winter  Fall  Spring | 30 (51)  25 (42)  3 (5) | 153 (68)  42 (19)  22 (10) | 0.002 |
| Temperature, °c  Chest pain  Cough  Running nose  Bacterial co-infection | 38.3 [37.4-39.0]  7 (13)  39 (71)  8 (17)  10 (19) | 38.4 [37.6-39.3]  29 (14)  135 (65)  11 (6)  61 (27) | 0.22  1.00  0.43  0.03  0.17 |
| ≥ 2 involved quadrants on chest X-ray  Interstitial aspect on chest X-ray  Ground glass on lung CT-scan  Diffuse alveolar aspect on lung CT-scan  Micronodular aspect on lung CT-scan | 44 (92)  34 (87)  20 (57)  10 (29)  12 (34) | 159 (85)  116 (91)  61 (58)  26 (25)  27 (26) | 0.42  0.62  0.98  0.78  0.45 |
| Neutropenia | 18 (31) | 84 (38) | 0.36 |
| Platelet count (10^9/L) | 140 [29-284] | 112 [38-175] | 0.38 |
| PaO_2_/FiO_2_ on day 1, mmHg | 157 [108-180] | 122 [90-173] | 0.004 |
| Oxygenation strategy on day 1 |  |  | 0.99 |
| NIV | 18 (31) | 64 (29) |  |
| High-flow oxygen  Invasive mechanical ventilation | 18 (31)  15 (26) | 71 (32)  69 (31) |  |
|  |  |  |  |
| SOFA score | 5 [4-8] | 6 [4-9] | 0.22 |
| Invasive mechanical ventilation (overall) | 20 (34) | 94 (42) | 0.30 |
| Vasopressor support | 19 (32) | 133 (59) | <0.001 |
| RRT | 9 (15) | 44 (20) | 0.57 |
| Invasive mechanical ventilation duration, days | 11 [3-33] | 10 [4-19] | 0.70 |
| ICU length of stay, days  Hospital length of stay, days | 7 [4-24]  18 [14-30] | 9 [4-17]  15 [10-29] | 0.60  0.62 |
| In-ICU mortality | 12 (20) | 72 (32) | 0.08 |
| In-Hospital mortality | 14 (25) | 85 (38) | 0.063 |

Note that 3 patients with both RSV and Influenza infection have been excluded

**Table S4**. Description of documented bacterial co-infections

| **Identified microorganisms** | **Overall**  **N = 79**  N (%) |
| --- | --- |
| **Gram positive** | **44 (56)** |
| *Streptococcus pneumoniæ*  *Staphylococcus Aureus* | 27 (34)  14 (18) |
| *Streptococcus group A* | 3 (4) |
| **Gram negative** | **35 (44)** |
| *Haemophilus influenzae* | 11 (14) |
| *Pseudomonas aeruginosa* | 8 (10) |
| *Klebsiella pneumoniae* | 7 (9) |
| *Escherichia coli* | 5 (6) |
| *Moraxella catharalis* | 4 (5) |

**Table S5**. Clinical characteristics, risk factors, and results from investigational procedures in 23 patients with virus detected respiratory failure and documented invasive pulmonary aspergillosis

|  | **N=23**  **N (%)** |
| --- | --- |
| **Patients characteristics** |  |
| Underlying disease  Allo-BMT  AML  Lymphoid disease  Solid tumor  SOT/drugs | 4 (17)  4 (17)  9 (39)  3 (13)  3 (13) |
| Steroids use | 17 (74) |
| Neutropenia | 8 (35) |
| **Chest CT-scan**  Focal alveolar pattern  Nodular lesions | 18 (78)  5 (22) |
| **Blood samples**  Galactomannan (blood) positive  PCR aspergillus (blood) positive | 19 (83)  4 (17) |
| **Respiratory samples**  Bronchoalveolar lavage  Galactomannan (BAL) positive  PCR aspergillus (BAL) positive  Aspergillus positive culture in respiratory sample:  Bronchoalveolar lavage  Others | 18 (78)  4 (17)  1 (4)  10 (43)  6 (26) |

BAL: fiberoptic bronchoalveolar lavage

**Figure S4**. Crude mortality rate according to virus species


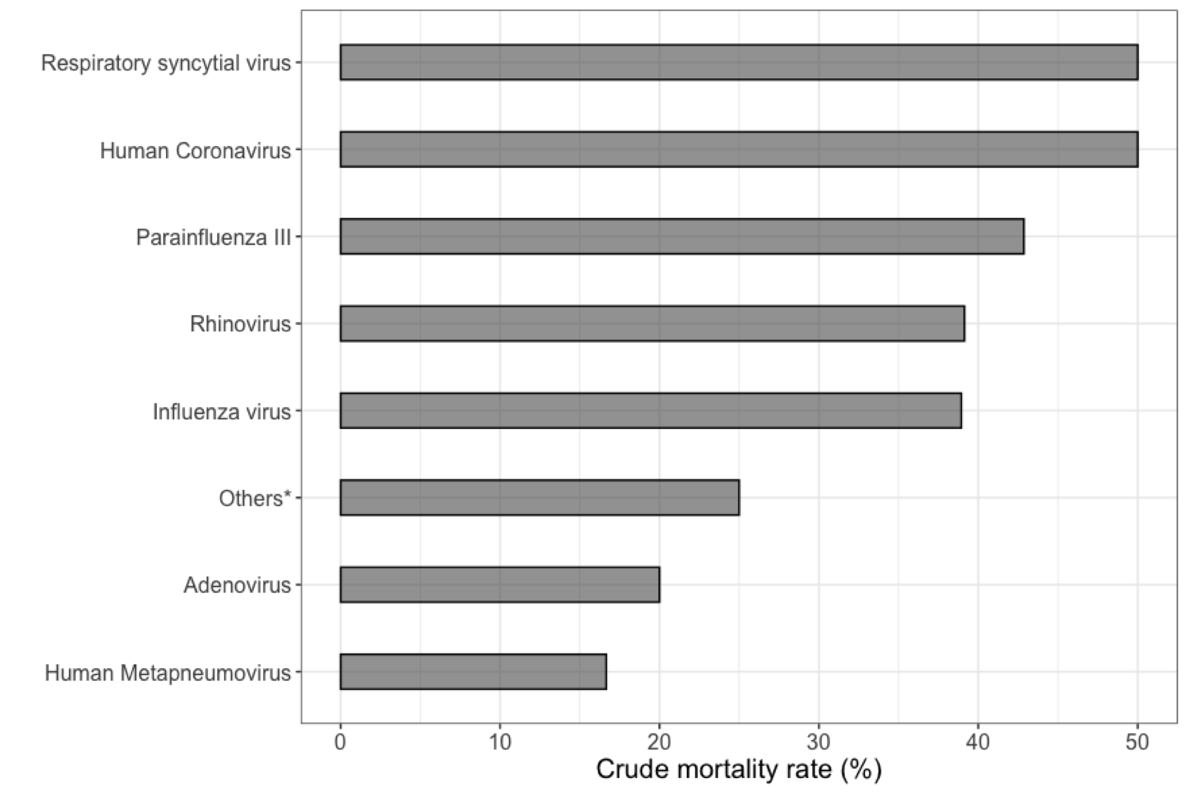


**3-Matched comparison of critically-ill immunocompromised patients with viral-ARF and a control group with ARF from other etiologies**

**Figure S5**. Absolute standardized mean difference between patients with and without virus-associated acute respiratory failure, before and after matching.


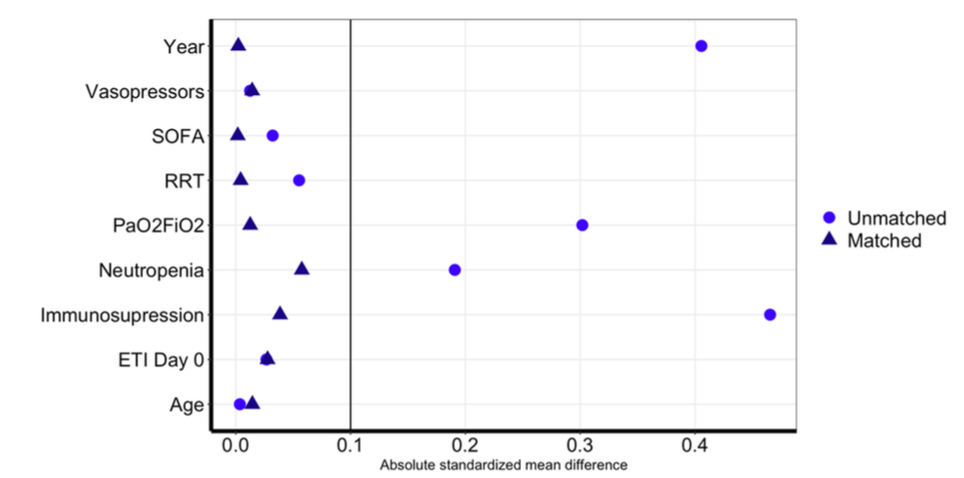


Absolute standardized mean difference is the absolute value of the difference in mean between groups divided by standard deviation. An absolute standardized mean difference of less than 0.2 usually shows a balance between groups.

PaO2/FiO2: Partial pressure of oxygen divided by the fraction of inspired oxygen; RRT: renal replacement therapy; SOFA: Sequential Organ Failure Assessment

**4-Sensitivity analysis**

**Table S6**. Pair matched odds ratio for hospital mortality according to diagnosis category, taking into account co-infections

|  | **OR [95%CI]** | **p-value** |
| --- | --- | --- |
| **Bacterial infection** | Reference level | - |
| **Detected virus** |  |  |
| Other respiratory viruses | — |  |
| Influenza | 0.92 [0.64-1.32] | 0.64 |
| Influenza-like* | 0.48 [0.27-0.85] | 0.01 |
| Co-infections viral-bacterial | 0.93 [0.58-1.52] | 0.78 |
| Co-infections viral-aspergillosis | 1.41 [0.60-3.34] | 0.43 |
| **Specific lung diseases** | 1.12 [0.83-1.50] | 0.45 |
| **Invasive fungal infection** | 2.05 [1.32-3.20] | <0.001 |
| **Pneumocystis pneumonia** | 1.24 [0.83-1.84] | 0.29 |

**Table S7.** Pair matched odds ratio for hospital mortality according to diagnosis category after excluding patients with bacterial pneumonia

|  | **OR [95%CI]** | **p-value** |
| --- | --- | --- |
| **Specific lung diseases** | Reference level | - |
| **Detected virus** |  |  |
| Other respiratory viruses | 0.98 [0.54-1.76] | 0.95 |
| Influenza | 0.91 [0.63-1.32] | 0.61 |
| Influenza-like* | 0.49 [0.28-0.87] | 0.01 |
| **Specific lung diseases** | 1.12 [0.83-1.50] | 0.45 |
| **Invasive fungal infection** | 2.44 [1.64-3.63] | <0.001 |
| **Pneumocystis pneumonia** | 1.11 [0.78-1.57] | 0.57 |

**Table S8**. Factors associated with in-hospital mortality in 370 critically ill immunocompromised patients with virus-detected acute respiratory failure taking into account co-infections

|  | **OR [95%CI]** | **p-value** |
| --- | --- | --- |
| **Detected virus** |  | 0.150 |
| Other respiratory viruses | Reference level |  |
| Influenza | 0.92 [0.45-1.88] | 0.824 |
| Influenza-like* | 0.51 [0.21-1.21] | 0.126 |
| Co-infections viral-bacterial | 0.86 [0.38-1.92] | 0.710 |
| Co-infections viral-aspergillosis | 1.36 [0.43-4.26] | 0.599 |
| **Neutropenia at admission** | 1.74 [1.07-2.81] | 0.025 |
| **Invasive mechanical ventilation,**  **day of admission** | 2.04 [1.19-3.50] | <0.001 |
| **Performans status** ≥ **2** | 1.79 [1.11-2.87] | 0.016 |

**References**

1. Biard L, Darmon M, Lemiale V, et al (2019) Center Effects in Hospital Mortality of Critically Ill Patients With Hematologic Malignancies. Crit Care Med 47:809–816. https://doi.org/10.1097/CCM.0000000000003717

2. Dumas G, Demoule A, Mokart D, et al (2019) Center effect in intubation risk in critically ill immunocompromised patients with acute hypoxemic respiratory failure. Crit Care Lond Engl 23:306. https://doi.org/10.1186/s13054-019-2590-7

3. Biard L, Porcher R, Resche-Rigon M (2014) Permutation tests for centre effect on survival endpoints with application in an acute myeloid leukaemia multicentre study. Stat Med 33:3047–3057. https://doi.org/10.1002/sim.6153
